# Temporal fluctuations in the sero-prevalence of *Taenia solium* cysticercosis in pigs in Mbeya Region, Tanzania

**DOI:** 10.1186/s13071-014-0574-7

**Published:** 2014-12-04

**Authors:** Uffe Christian Braae, Pascal Magnussen, Faustin Lekule, Wendy Harrison, Maria Vang Johansen

**Affiliations:** Section for Parasitology and Aquatic Diseases, Department of Veterinary Disease Biology, Faculty of Health and Medical Sciences, University of Copenhagen, DK-1870 Frederiksberg, Denmark; Institute for International Health, Immunology and Microbiology, Centre for Medical Parasitology, University of Copenhagen, DK-1353 Copenhagen, Denmark; Faculty of Agriculture, Sokoine University of Agriculture, Morogoro, Tanzania; Faculty of Medicine, School of Public Health, Imperial College London, London, UK

**Keywords:** *Taenia solium*, Porcine cysticercosis, Sero-prevalence, Tanzania, Fluctuations

## Abstract

**Background:**

Porcine cysticercosis is an emerging agricultural problem in sub-Saharan Africa. This has been documented primarily through cross-sectional studies, however detailed knowledge of the transmission dynamics of this disease in sub-Saharan Africa is lacking. This study aims to describe seasonal variations in sero-prevalence of antigen ELISA positive porcine cysticercosis in an endemic area.

**Methods:**

A longitudinal study composed of three cross-sectional surveys was carried out in Mbeya Region, Tanzania; the first two six months apart (March/April 2012 and October/November 2012) and the last eight months later (July/August 2013). Venous blood was collected from pigs in 22 villages and analysed using Ag-ELISA.

**Results:**

In each survey between 800–1000 serum samples were collected. The first survey revealed a cysticercosis sero-prevalence of 15% (n = 822, 95% CI: 13-18%). The sero-prevalence had significantly increased to 24% (p < 0.001, χ2-test, n = 812, 95% CI: 21-27%) at the time of the 6 month follow-up. At 14-months the sero-prevalence had dropped to 20% (p = 0.053, χ2-test, n = 998, 95% CI: 18-23%). Overall, this was a reduction in sero-prevalence compared with a study conducted in 2007 in the same area, where 31% (186/600) of pigs were found positive.

**Conclusion:**

Confined pigs did not have a lower sero-prevalence compared to free roaming pigs in any of the three surveys. Several factors may have contributed to the observed fluctuations such as African swine fever or seasonal variation in local crop production practices. Also, as the Ag-ELISA assay used is not species specific, variation in transmission of *Taenia hydatigena* could potentially influence the results. The observed fluctuations contradict a theoretical model which predicts a stable equilibrium, which only considers a two-compartment (pig and human) model excluding the effect of the environment. Whether the disease has an endemic equilibrium, or undergoes fluctuations dependent on extrinsic and/or socio-economic factors remains to be elucidated.

## Background

The zoonotic tapeworm infection *Taenia solium* taeniosis/cysticercosis has in the past decades emerged as a serious agricultural and public health problem in sub-Saharan Africa [1,2]. Porcine cysticercosis has significantly impacted pig production in sub-Saharan Africa, by reducing the market value of infected pork, resulting in economic losses for farmers [3,4]. Pigs are highly proliferative and have the ability of converting otherwise wasted resources such as kitchen leftovers and unused crop products into dietary protein, and therefore, of vital importance in alleviating the challenges of food security. However, food safety is an emerging problem along with *T. solium* taeniosis/cysticercosis.

Understanding the epidemiology and transmission dynamics of *T. solium*, and the possible variation from region to region is essential if control strategies are to be successfully implemented and sustainable on a large scale. Despite decades of research, fundamental questions about the epidemiology and transmission of *T. solium* remain unanswered.

Very few directly comparable studies are currently available. A study carried out in Peru over a nine month period, with assessments preformed at approximately three monthly intervals reported prevalence of porcine cysticercosis based on Enzyme-linked Immunoelectrotransfer Blot (EITB) of 61%, 57%, and 66%, respectively [5]. In Nigeria, a study reported higher prevalence of porcine cysticercosis based on post-mortem inspection during the rainy season, but figures were not significantly different compared with the rest of the year. However a significant drop was reported over a three year period [6].

A theoretical model simulating the transmission of *T. solium* in an endemic area predicts a stable level of porcine cysticercosis endemicity over time [7]. However, the model only operates with a two-compartment scenario (man and pig) and does not take into account that eggs can be viable in the environment for an extended period of time [8]. Also, because pigs are highly proliferative and have a relative short lifespan, fluctuations in prevalence related to extrinsic factors can be expected to occur. This study aimed at investigating sero-prevalence over time and describes factors that could possibly influence fluctuations in porcine cysticercosis prevalence.

## Methods

### Study area

The study was conducted in Mbeya Region, Tanzania, in the two districts Mbeya and Mbozi located between latitudes 8°14′ and 9°24′S and longitudes 32°04′ and 33°49′E. The climate is subtropical with the rainy season lasting from November to May, with the majority of rainfall between December and March. The human population was estimated in 2012 by census to be 305,319 in Mbeya district and 446,339 in Mbozi district [9]. Both districts are rural areas where pig production is almost exclusively on a smallholder level, with 31,190 pigs in Mbeya district and 117,483 pig in Mbozi district in 2007/2008 [10]. Pigs that are not confined throughout the year are more likely to be confined between November and June and allowed to roam free for the rest of the year.

### Study design and sample size

**Figure 1:**
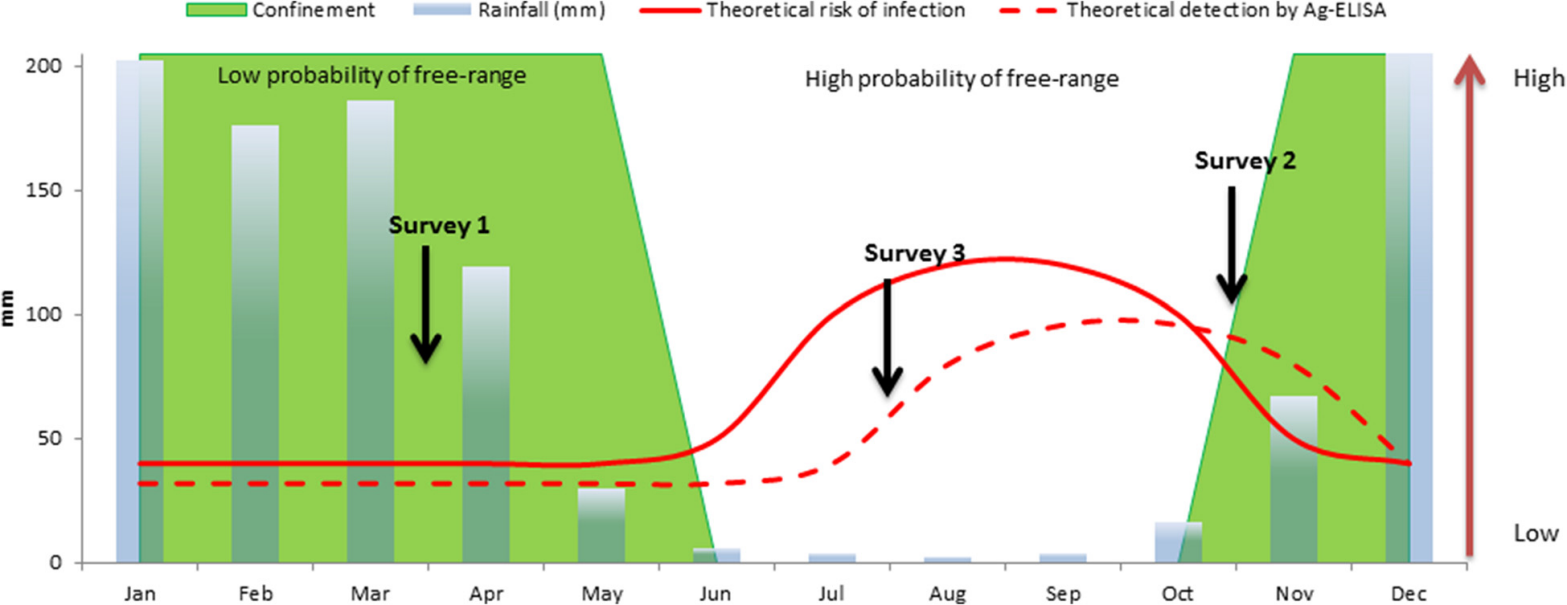
**Theoretical risk of infection based on pig production system.** The theoretical risk of infection is set to arbitrary values from low to high based on the likelihood of free-range being practised, which is based on rainfall in Mbeya Region that determines planting seasons and therefore when free-range is practised. The theoretical detection of infection is based on risk of infection and a test sensitivity of 80% with one month lag due to the 2–6 weeks it takes for antigen levels to reach detectable levels in infected pigs. The three surveys are placed according to the time of year they were carried out and not in chronological order.

A longitudinal study was carried out consisting of three cross-sectional surveys; the first two executed approximately six months apart, and the last approximately eight months later. The first survey was performed during the rainy season in March and April 2012, and the 6-month follow-up survey was performed towards the end of the dry season in October and November 2012. The final 14-month follow-up survey was carried out in the dry season in July and August 2013 (Figure 1). In each district, 11 villages were included in the study based on known presence of porcine cysticercosis [11]. From each village, all non-pregnant pigs more than 2-month-old were included in the study. However, before the 14-month follow-up the pig population had increased to a size so it was no longer feasible to sample all pigs. Thus, a maximum of four pigs were selected at random from each pig keeping household using a simple lottery method.

### Data collection and Ag-ELISA analysis

Production systems were classified as either confined (pigs kept in a pen), semi-confined (tethered), or free-range (free roaming). Type of production system was noted based on what could be observed at the time of visit. The sex and age of pigs was recorded before blood sampling. From each pig 10 ml of jugular vein blood was drawn, centrifuged and serum aliquoted in two 1.8 ml cryogenic vials on the day of collection. Serum samples were kept at −20°C prior to analysis. Serum samples were analysed for circulating porcine cysticercosis antigens (Ag) at the University of Zambia in Lusaka using an Ag-ELISA assay as described by Brandt *et al*. [12] and modified by Sikasunge *et al*. [13]. The assay is reported to be genus specific, and not species specific and have an approximate sensitivity of 87% and a specificity of 95% [14].

### Ethical considerations

The study was approved by the Imperial College Research Ethics Committee (ICREC), reference no. ICREC_11_3_6. Permission to conduct the study was sought through Sokoine University of Agriculture in Morogoro, Tanzania, and regional, district, and local village authorities. All animals were handled in strict accordance with good animal practice as defined by the OIE’s Terrestrial Animal Health Code for the use of animals in research and education. Oral consent for porcine blood collection was also sought from all participating farmers after they were informed about the aim, risks, and benefit of the study. Pigs found positive for *T. solium* cysticercosis, were treated at the subsequent visit with oxfendazole (30 mg/kg) using an oral drench gun with Synanthic 9.06 oral suspension, Merial, France, (batch no. F10701B). Owners of treated pigs were told that the pork was unfit for human consumption if the pig was slaughtered within 28 days of treatment. This information was also given to them in writing. Farmers were also informed about ways to improve pig management and prevent *T. solium* cysticercosis.

### Statistical methods

All data was entered into an Excel spread sheet (Microsoft Office Excel 2010®) and imported into the statistical programme R (http://www.r-project.org). Sero-prevalence and 95% confidence intervals (95% CI) were calculated and the difference in sero-prevalence between the three surveys was analysed using χ^2^-tests. Logistical regression with porcine cysticercosis as the dependent variable was performed for each survey to control for association with the independent variables; production system, sex, and age. Odds ratios (OR) were calculated for all independent variables. Mean differences in age was explored with One-Way ANOVA & Tukey test.

## Results

**Figure 2:**
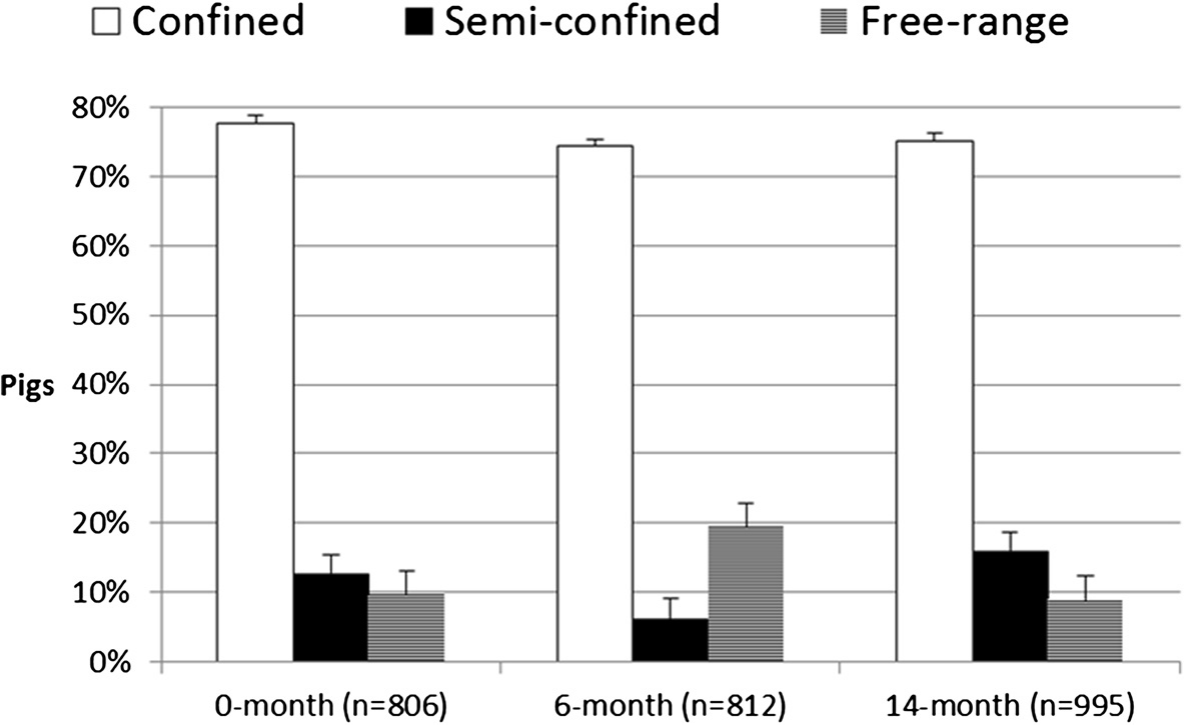
**Proportion of pigs kept under each specific production system during each sampling round.** Sample size = n. Error bars represent the standard error in percentage points.

Pigs were sampled from 450 households with serum collected from a total of 822 pigs in the first survey. Porcine production and population demographics from each of the surveys are listed in Table 1. There was a significant increase in mean age within the pig population during the study from first to last survey (P > 0.0001, One-Way ANOVA & Tukey). The production system illustrated in Figure 2 was recorded for all pigs, except for 16 pigs in the first survey and 3 pigs in the third survey.

**Table 1:** **Household and porcine data collected from three surveys in Mbeya and Mbozi districts, Mbeya Region, Tanzania**

	**Baseline**	**6-month follow-up**	**14-month follow-up**
**Time of survey**	**March/April 2012**	**October/November 2012**	**July/August 2013**
Number of Households	450	424	511
Number of pigs	822	812	998
Male %	41	37	37
Female %	59	63	63
Age in month (mean)	7.38 (±4.58^a^)	8.89 (±5.97^a^)	9.48 (±6.04^a^)
Production system practised			
Confined (%)	627 (77.8)^b^	604 (74.4)	748 (75.2)^c^
Semi-confined (%)	101 (12.5)^b^	50 (6.2)	158 (15.9)^c^
Free-range (%)	78 (9.7)^b^	158 (19.5)	89 (8.9)^c^
Porcine cysticercosis			
Number of positive pigs^d^	127	198	202
Sero-prevalence % and (95% CI)	15 (13–18)	24 (21–27)	20 (18–23)
Difference between surveys	NA	<0.001^e^	0.053^e^

^a^± Standard deviation.

^b^Based on 806 pigs – production system was not record for 16 pigs (n = 822).

^c^Based on 995 pigs – production system was not record for three pigs (n = 998).

^d^Based on Ag-ELISA.

^e^P values for χ^2^-test comparison between the preceding survey.

**Figure 3:**
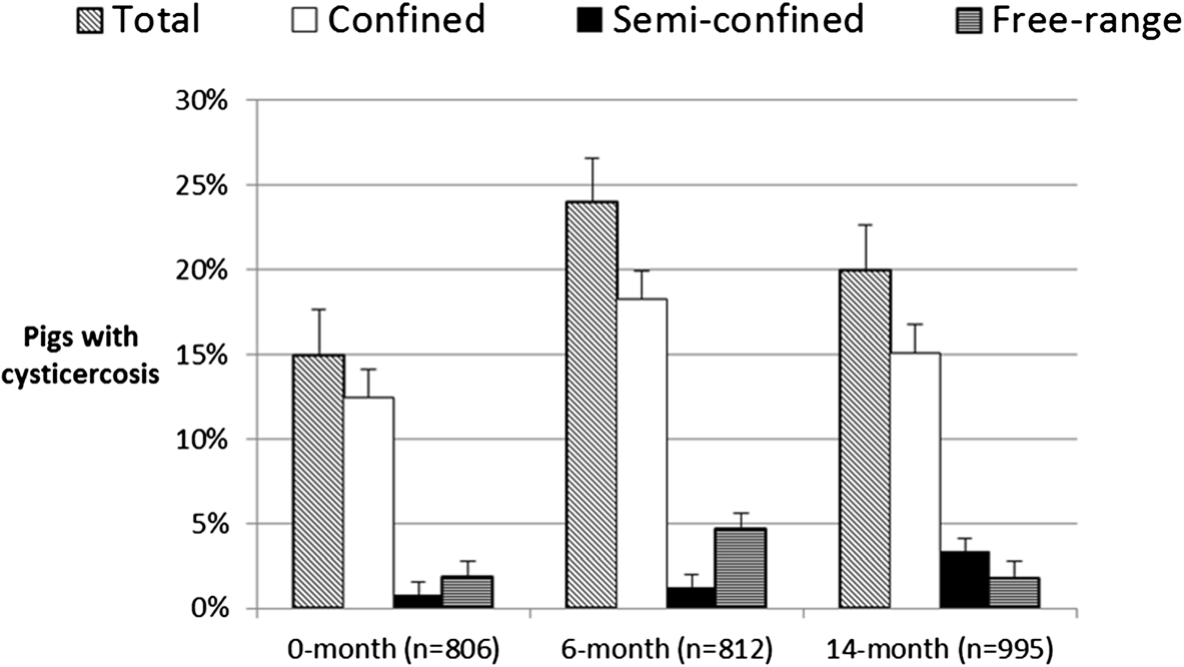
**Overall sero-prevalence of porcine cysticercosis and sero-prevalence within each production system with total number of pigs as the denominator.** Sample size = n. Error bars represent the standard error in percentage points.

**Figure 4:**
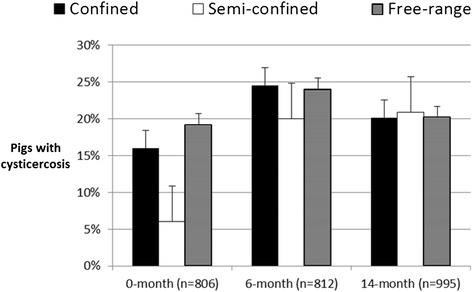
**Sero-prevalence of porcine cysticercosis measured for each type of production system with number of sampled pigs from the respective production system as the denominator.** Sample size = n. Error bars represent the standard error in percentage points.

In the first survey the sero-prevalence of porcine cysticercosis was 15% (n = 822, 95% CI: 13-18%). In the second survey the sero-prevalence had significantly increased to 24% (p < 0.001, χ^2^-test, n = 812, 95% CI: 21-27%). In the third survey the sero-prevalence had dropped to 20% (p = 0.0531, χ^2^-test, n = 998, 95% CI: 18-23%) which was borderline significant (Table 1). Sero-prevalence of porcine cysticercosis for each type of production system, with total number of sampled pigs as the denominator, is illustrated in Figure 3. Most positive pigs were from the confined production system, but the majority of pigs were also sampled from the confined production system. Figure 4 depicts the within production system sero-prevalence for each of the three rounds. It is clear that there is negligible difference between the production systems except for semi-confinement during the first survey which has significantly lower sero-prevalence. Logistic regression showed that in the first survey there was no difference between positive pigs from confined and free-range production systems, but semi-confined pigs were significantly (P = 0.0194) less likely to be infected with *T. solium* with an OR of 0.35 (95% CI: 0.14-0.78) compared to confined pigs. Borderline association between female pigs and infection was found (OR = 1.51, 95% CI: 0.99-2.33, P = 0.0604) with slightly higher risk for females compared to males. In the second and third surveys neither production system nor sex, could be associated with the sero-prevalence of porcine cysticercosis, but in the last survey age was associated with increased risk of infection (OR = 1.02, 95% CI: 1.01-1.06, P = 0.0111). Data from all three surveys confirmed that confinement, under the settings that were present in Mbeya Region, did not prevent pigs from becoming infected with porcine cysticercosis.

## Discussion

Several factors may have contributed to fluctuations in the observed sero-prevalence of porcine cysticercosis. In December 2010, prior to the surveys, the study area suffered an outbreak of African swine fever (ASF) which decimated the regional pig population of 346,466 [10] with 10,240 reported deaths (Ministry of Livestock and Fisheries Development). Pigs being kept under free-range conditions is considered to be one of the main risk factors for the spread of ASF in certain areas [15,16] and also identified as an important risk factor for porcine cysticercosis [13,17-19]. Therefore the outbreak might have changed the pig production systems predominantly practised which could affect the transmission dynamics of *T. solium*. During the end of the outbreak, the enforcement of community restrictions implemented at village-level, deterring farmers from keeping pigs under free-range conditions, may have led to an increase in the number of pigs being confined. In support of this Komba *et al.* [11] reported that in 2007–2008 only 42% of the sampled pigs within the study area were confined and 58% were either semi-confined or kept under free-range conditions. However, these numbers might not reflect the true situation since they are reported on household level and not on pig level, and many farmers will not practise one production system exclusively at any one time. The results from the study by Komba *et al.* [11] do differ significantly from those reported in this study where approximately three quarters of the pig population were confined.

In Mbeya Region farmers often switch between production systems depending on the agricultural season. If pigs are not confined throughout the year, they are usually free-ranged from June to November. Seasonal variation in the prevalence of porcine cysticercosis could exist because of these different seasonal production systems, possibly leading to higher transmission from June to November when more pigs are free-ranged and a low transmission season from December to May when more pigs are kept confined to prevent their destruction of planted crops on the fields. However, production system was not associated with infection in this study except for the decrease risk of infection with the use of semi-confinement in the first survey. Furthermore, the proportion of pigs kept confined was relatively constant (74-78%) during this study.

The high porcine cysticercosis sero-prevalence recorded in this study is surprising since three quarters of the pig population was confined at each time of surveying. This could indicate that confinement does not prevent porcine cysticercosis, and thus, insufficient as a sole intervention measure for preventing porcine cysticercosis in the studied districts. More research is needed to investigate whether transmission takes place inside the pens or if pigs are infected outside the pens.

Estimating porcine cysticercosis sero-prevalence is further complicated by the Ag-ELISA assay which is not species specific [12,14]. Therefore variation in the prevalence of *T. hydatigena* could influence the sero-prevalence estimation of porcine cysticercosis when measured by Ag-ELISA. There is no documentation of seasonal variation in the transmission of *T. hydatigena*. A previous study carried out in the northern part of Tanzania reported a *T. hydatigena* prevalence of 1.4% in pigs [20]. However, to what extent pigs are infected in the southern part of Tanzania is currently unknown.

Age was not associated with the risk of porcine cysticercosis in the first two surveys, but was a significant risk factor in the third survey. The mean age within the pig population was increasing throughout the study, suggesting an increase per pig exposure time throughout the study period.

The fluctuations observed over time are in contradiction with the theoretical compartment model which predicts a stable equilibrium of pigs infected with *T. solium* [7]. However, the model is only based on two-compartments (pig and man) and therefore lacks the environment as a compartment. The model is based on a one year average life span of pigs. If *Taenia* eggs, which can stay viable within the environment for extended periods [8], survive in the environment for more than 12 months, the current model is insufficient and will have to be modified to yield a more realistic transmission scenario.

Whether the prevalence of cysticercosis will further increase and return to the recorded levels found in the Mbeya Region in 2007–2008 before the outbreak of ASF, and if that level can be characterised as the equilibrium, is unknown. However the results obtained in this study suggest that fluctuations will continue to occur. Further studies are needed to determine if porcine cysticercosis has an endemic equilibrium, or in fact go through fluctuations with or without the presence of the factors described in this paper. Identifying and understanding possible factors responsible for fluctuations of porcine cysticercosis will be essential when designing integrated control programmes for *T. solium* and for modelling transmission breakpoint.

## Conclusion

The present study demonstrated a significant temporal fluctuation in the sero-prevalence of *T. solium* cysticercosis in pigs and our conclusion was further supported by findings from a previous cross-sectional study from the same area using the same diagnostic method [11]. The study also found that confinement of pigs did not reduce the sero-prevalence of porcine cysticercosis in Mbeya Region, Tanzania. Interestingly, with the large proportion of confined pigs being infected, this study indicates that these pigs are either not truly confined or infection takes place within the pen, and could therefore be related to environmental contamination of *Taenia* eggs. Control focusing solely on pigs or the human carriers might not be effective if pigs are infected indirectly from the environment. Control will therefore require a comprehensive One Health approach over a prolonged period.
